# Parsimony, not Bayesian analysis, recovers more stratigraphically congruent phylogenetic trees

**DOI:** 10.1098/rsbl.2018.0263

**Published:** 2018-06-20

**Authors:** Robert S. Sansom, Peter G. Choate, Joseph N. Keating, Emma Randle

**Affiliations:** 1 School of Earth and Environmental Sciences, University of Manchester, Manchester M13 9PT, UK; 2 Faculty of Biology, Medicine and Health, University of Manchester, Manchester M13 9PT, UK; 3 Department of Biology and Biochemistry, University of Bath, Bath BA2 7AY, UK

**Keywords:** morphology, phylogeny, parsimony, stratigraphic congruence, Bayesian

## Abstract

Reconstructing evolutionary histories requires accurate phylogenetic trees. Recent simulation studies suggest that probabilistic phylogenetic analyses of morphological data are more accurate than traditional parsimony techniques. Here, we use empirical data to compare Bayesian and parsimony phylogenies in terms of their congruence with the distribution of age ranges of the component taxa. Analysis of 167 independent morphological data matrices of fossil tetrapods finds that Bayesian trees exhibit significantly lower stratigraphic congruence than the equivalent parsimony trees. As such, taking stratigraphic data as an independent benchmark indicates that parsimony analyses are more accurate for phylogenetic reconstruction of morphological data. The discrepancy between simulated and empirical studies may result from historic data peaking practices or some complexities of empirical data as yet unaccounted for.

## 1. Introduction

Phylogenetic trees are vital for reconstructing evolutionary events. Incorporating morphology and fossils into phylogenetic analyses makes it possible to break up long branches between living taxa, identify the sequences of events in the construction of body plans, and reconstruct timescales and rates of evolution by calibrating molecular clocks. Accurate phylogenetic placement is, therefore, vital for reconstructing evolutionary history, but there is disagreement over which method is most appropriate. Morphological data have traditionally been analysed using maximum parsimony but recent investigations have indicated that probabilistic methods outperform parsimony analyses in terms of accuracy [1,2] (although see [3]). As such, Bayesian analysis (specifically the Mk model) is suggested to be the preferred method for analysing morphological data [4]. Simulated data have been extremely powerful in this context, but simulations can only ever approximate reality with varying levels of success. Empirical morphological data are highly variable and intrinsically problematic in complex and unsimulated ways. Homoplasy and non-independence of morphological characters are pervasive due to subjective and oversaturated character identification, and functional, ecological and developmental linkage [5–8]. These problematic phenomena are not evenly distributed across clades, characters and regions; differences exist between hard and soft characters [9,10], teeth and bones [11], and crania and post-crania [12]. Furthermore, non-random patterns of missing data can distort the signal of remaining phylogenetic characters [9,13,14]. By contrast, simulated characters are effectively homogeneous, because they are selected from the same underlying model.

Here, we use empirical data to evaluate parsimony and Bayesian-derived phylogenies. This enables inclusion of the intrinsic complexities and weaknesses of real data, but problematically, the underlying true phylogeny is unknowable. We use congruence with stratigraphic range data as an independent benchmark. If the fossil record were perfect, then the appearance of fossil taxa through time should match their branching order in a phylogeny (i.e. the first occurrences of taxa from early branching lineages are in older rocks while later branching taxa are more recent). Congruence between phylogenies and stratigraphic ranges of component taxa has been observed using a range of different metrics and taxa [15–17], but stratigraphic data can be problematic due to preservation biases and incompleteness of the geological record [18]. Given different topologies for the same set of fossil taxa, stratigraphic congruence metrics can be used to support one solution or technique over another (e.g. [19]), but broader conclusions will be hard to draw from any one dataset. Here, we take a meta-analysis approach; we use a wide range of published morphological data matrices of crown-group tetrapods to compare maximum parsimony (equal and implied weights) and Bayesian (Mk) trees in terms of stratigraphic congruence. By including data from a wide range of clades, authors and time periods, any significant patterns that result are likely due to intrinsic properties of the data and analyses rather than spurious correlation. As such, we use stratigraphic congruence to test techniques of phylogenetic reconstruction using morphological data.

## 2. Material and methods

We used published morphological matrices of crown-group tetrapods (*n* = 2177) from the online repository of Graeme Lloyd [20]. Taxon age ranges (first and last known ages of occurrence of fossil species, genus, family, etc.) were retrieved from the palaeobiology database (electronic supplementary material). Datasets that did not meet our minimum criteria or were not amenable to analysis in MrBayes [21] were excluded (electronic supplementary material). To avoid excessive taxonomic overlap and non-independence of datasets, matrices were sequentially eliminated to ensure a minimum of 50% unique taxa in each matrix, and an average of 75% unique taxa for all matrices (electronic supplementary material).

Parsimony searches were conducted in TNT [22] with equal and implied character weighting (*k* = 3, 12) [23]. Bayesian searches were conducted in MrBayes [21] using the standard models of morphological analyses [4] with ‘informative’ ascertainment bias as the morphological datasets were originally conceived for parsimony analyses (Mki). Across site rate variation was drawn from a gamma distribution (electronic supplementary material). The R package strap [16] was used to derive SCI (Stratigraphy Consistency Index [24]), RCI (Relative Completeness Index [25]), GER (Gap Excess Ratio [26]) and MSM* (modified Manhattan Stratigraphic Measure [27]) values for the most parsimonious trees and trees from the posterior Bayesian distribution (maximum subsample of 500 for each), not consensus trees. The metrics use the proportion of tree nodes that are stratigraphically congruent (SCI), or various relationships between the sum of unobserved ghost ranges and sum of observed ranges (RCI), or minimum possible ghost ranges (MSM*) and maximum possible ghost ranges (GER). Tree samples were tested for significant stratigraphic fit in STRAP using 1000 random permutations and 1000 sampled permutations.

## 3. Results

**Figure 1.**
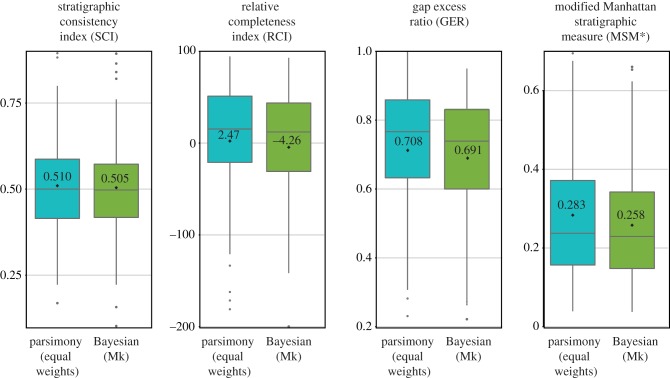
Distribution of average stratigraphic measures of trees from 167 datasets (either most parsimonious trees with or trees sampled from the posterior distribution of Bayesian searches). Box plots show median, upper and lower quartiles and outliers, while black spots and text values are averages for all datasets. (Online version in colour.)

The final data sample of 167 data matrices comprised 5719 operational taxonomic units with stratigraphic range data, of which 4230 were unique. The resulting trees showed high levels of stratigraphic congruence for all metrics and all methods (Fisher's combined probability of randomization tests for individual matrices *p* < 1 × 10^−10^), which accords with [15]. Of the different tree search techniques, the Bayesian trees exhibited lower average stratigraphic congruence (figure 1; electronic supplementary material, figure S1) and less frequently recovered significant stratigraphic congruence (electronic supplementary material, figure S2). The differences between search methods are significant for all four congruence metrics (*p* < 2.2 × 10^−16^ in linear mixed-effect models with repeated measures for data matrix and accounting for variable variances). Adding the presence or the absence of a tree figure optimized to stratigraphic ranges in the original publication as an explanatory variable (Strat_Fig) increased the fit of the linear mixed-effect models (ANOVA *p* < 0.0001 for all four metrics); this variable was not significant (except GER), but its interaction with tree search method was (*p* < 2 × 10^−16^ for all four metrics). Nevertheless, the interaction does not occur in the direction such that equal-weight parsimony trees derived from studies that had stratigraphic range trees present exhibited elevated congruence relative to other combinations.

Comparing average stratigraphic congruence metrics of equal weight parsimony trees and Bayesian trees (electronic supplementary material, figure S3) found that data matrices with higher stratigraphic congruence showed an elevated tendency to have a higher congruence of equal-weight parsimony trees relative to Bayesian trees (linear regression slope values were significantly lower than 1 for SCI, GER and MSM* (*p* < 1 × 10^−5^).

## 4. Discussion

Bayesian analyses yielded trees that were significantly less congruent with stratigraphic data. Given that the 167 empirical datasets were from a wide range of authors, clades, time periods and taxonomic levels, we can place confidence in the small but significant differences observed. Taking stratigraphic range data as a benchmark independent of morphology, therefore, indicates that parsimony should be preferred over Bayesian analyses, but these empirical results differ from simulation studies. We explore a few possible explanations for this discrepancy.

First, congruence metrics might be problematic or afflicted by stratigraphic biases. The levels of empirical congruence found here are generally high, however (see also [15]), and there are few reasons to expect that any one phylogenetic reconstruction technique would be more biased than others for all stratigraphic metrics. Bayesian searches yield a higher number of trees than parsimony searches (the posterior distribution) and are thus inherently less precise than most parsimony searches, but they are found here to also be less accurate in terms of stratigraphic congruence; parsimony searches were comparatively both more precise (fewer trees) and more congruent with stratigraphic range data.

Second, it is possible that stratigraphic range data and phylogenetic data may not be strictly independent. Cycles of revision and re-analysis of morphological data matrices during construction could lead practitioners to prioritize phylogenetic solutions that fit some preconceived ideas for final publication (either consciously or subconsciously), including stratigraphic fit. Under such circumstances, parsimony trees might exhibit artificially elevated stratigraphic congruence because parsimony is the historic default method used to evaluate morphological data. Comparison of data matrices from studies that did, and did not, explicitly consider stratigraphy (i.e. included a tree figure optimized to age range data) found little evidence of bias or data peaking (equal-weight parsimony searches did not exhibit elevated congruence for those studies). On the other hand, datasets that exhibited higher stratigraphic congruence tended to exhibit higher congruence of parsimony trees relative to Bayesian trees (electronic supplementary material, figure S3). This is the pattern to be expected if parsimony results had been used to select solutions with higher stratigraphic congruence; nevertheless, the weak effect might be driven by other factors.

Third, previous simulations may have over-simplified the complexity of real-world data. To take account of character non-independence, subjectivity and non-random distributions of homoplasy, it is first necessary to identify the distribution and magnitude of these problematic phenomena in empirical data. Whether these factors account for the different findings from simulated and empirical data is unclear because the analyses also differ in the benchmarks used and other data properties. It is not immediately clear, however, that Bayesian analysis would relatively be more affected by these problematic phenomena.

Aside from previous simulation studies, there may also be limitations in Bayesian models available. The complexity of morphology is being increasingly accounted for by Bayesian models [20], and total-evidence tip-dating analyses enable incorporation of stratigraphic data directly into phylogenetic analyses [28]. Stratigraphic congruence could be used to prioritize topologies from the Bayesian posterior distribution of trees, but this would need balancing against levels of imprecision of Bayesian searches.

In conclusion, our analyses demonstrate a clear result: Bayesian searches yield trees that have significantly lower stratigraphic congruence compared with trees from parsimony searches. We find little difference between parsimony using equal and implied character weighting—they are roughly comparable with respect to stratigraphic congruence. If stratigraphic congruence is taken as a benchmark for phylogenetic accuracy, then, maximum parsimony is the preferred method of choice for analysis of morphological data.

## Supplementary Material

Supplementary Information

## Supplementary Material

Supplementary Table

## Data accessibility

The electronic supplementary material contains details of the methods and results. The datasets and age range data used in this study are deposited in the Dryad Digital Repository (http://dx.doi.org/10.5061/dryad.f9v3778) [29].
