# Supplementary material for: Parsimony, not Bayesian analysis, recovers more stratigraphically congruent phylogenetic trees
Source: Biol Lett. 2018 Jun 20;14(6):20180263. doi: 10.1098/rsbl.2018.0263 (PMC6030593; doi:10.1098/rsbl.2018.0263)
Supplement: Supplementary Information [file rsbl20180263supp1.docx]

**Parsimony, not Bayesian analysis, recoversr the most stratigraphically congruent phylogenetic Trees**

**Sansom *et al.* Biology Letters**

**http://dx.doi.org/10.1098/rsbl.2018.0263**

**Supplementary Information**

*Datasets-* Our starting sample was 2177 morphological data-matrices of crown-group tetrapods from Graeme Lloyd’s online repository (extracted June 2017, <http://www.graemetlloyd.com/matr.html>) [20]. Datasets that did not meet our criteria for the minimum number of taxa (10), characters (20), ratio of taxa to characters (2:3), or were not amenable to analysis in MrBayes [21] (10+ characters states, ordered characters 6+ states), were excluded. Matching age range data (first and last known occurrences) were sought for the individual taxa in the remaining datasets from the palaeobiology database (<https://paleobiodb.org/>) using publicly available API. If an exact taxon name match was found, this was accepted. Spelling mistakes and abbreviations were manually curated. For manually curated taxa, a match was accepted only if the spelling mistake was trivial or the abbreviation was elaborated in the paper. Other unmatched taxa, such as specimen numbers, extant non-fossil taxa, *nomen dubia*, unexplained abbreviations, non-trivial spelling mistakes, arbitrary groupings of taxa and taxa not present on paleobiodb.org were declared ‘missing’. 88% of taxon entries were matched to stratigraphic range information. Such taxa were retained in phylogenetic matrices for tree searches but excluded from stratigraphic congruence calculations.

For meta-analyses of empirical phylogenetic data, it is necessary to consider the independence of datasets. To avoid certain groups being represented in the data sample multiple times (e.g. inclusion of the same matrix multiple times that has been slighted edited in different publications), matrices were assessed in terms of taxonomic overlap. A maximum for the percentage of taxa in any one matrix duplicated in any other matrix was set at 50%, with average matrix uniqueness set at 75%. A duplication score (Di) was calculated for each matrix:


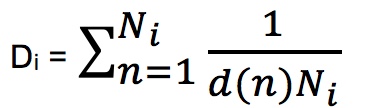


Where N_i_ is the number of taxa in matrix i, n is the n^th^ taxon in the matrix and d(n) is a function that returns the number of times that taxon n appears in all matrices that have not been eliminated. This returns a score between 0 (complete overlap) and 1 (entirely unique). The matrix with the lowest score was removed and then the scores were recalculated. If two matrices had the same score, the one with the most recent publication date was retained. This process was repeated until all matrices had at least 50% unique taxa with stratigraphic range information, with an average of 75% uniqueness across all matrices.

The final data sample of 167 data matrices comprised 5719 operational taxonomic units with stratigraphic range data, of which, 4230 were unique. These datasets had an average of 43 characters and 173 characters each. The included datasets were classified according to the presence or absence in the original publication of a figure of a tree optimised onto stratigraphic ranges (Strat_Fig). Outgroups were manually identified and stipulated for each dataset.

*Analyses*- Parsimony searches were conducted in TNT using both equal weighting and implied weighing (concavity constant *k*=3 or 12) of characters [22-23]. Searches used ‘new technology’ with tree-drifting, tree-fusing, and sectorial searches (*xmult: level 10*) and subsequent branch breaking (*bbreak*) retaining a maximum of most parsimonious 100,000 trees for each matrix. Bayesian searches were conducted in MrBayes under the Mk model with informative ascertainment bias (*lset coding=informative*) and gamma-shaped rate distribution (*lset rates=gamma*), using 5000000 generations, 2 runs of 4 chains, and a sampling frequency of 500 [4,21]. Searches were accepted as converged if ESS was greater than 200 and alpha PSRF was less than 1.1. The resulting trees from all searches were randomly subsampled to a maximum of 500 per search criterion (parsimony with equal weights, parsimony with implied weights, Bayesian analysis) for each matrix (excluding the 25% of burn-in trees for the Bayesian searches).

The resulting congruence metric scores were tested in mixed linear models in R, using individual scores for each tree and allowing variable variances between search methods (e.g. lme(GER~Method, random=~1|Dataset, weights=varIdent(form=~1|Method), method=”ML”). Four of the 167 datasets did not reach convergence in the Bayesian analysis (either ESS less than 200 or alpha PSRF more than 1.1), but their exclusion from the data sample did not change the qualitative outcome of the statistical tests. An alternative approach in which average stratigraphic congruence metrics for each dataset and each method are used rather than individual trees also finds significant differences for RCI, GER and MSM* (*p*<0.05 for one way ANOVAs with repeated measures) but not for SCI. The same pattern is found when results from only Bayesian and equal weighted parsimony are compared.

**Supplementary Figure 1**. Distribution of average stratigraphic measures of trees from 167 data sets analysed under parsimony (equal weights or implied weights *k*=3, or *k*=12) and Bayesian analyses. Box plots show median, upper and lower quartiles, outliers, whilst black spots and text values are averages for all datasets.


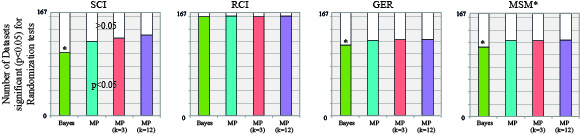


**Supplementary Figure 2.** Results of the randomisation tests find generally high levels of significant stratigraphic congruence (*p*<0.05), and show that Bayesian searches less frequently exhibit significant congruence than parsimony searches for SCI, GER and MSM* (Friedman test *p*= 8.3x10^-9^, 0.0015, 0.00053 respectively).


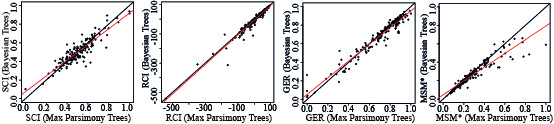


**Supplementary Figure 3.** Comparison of average dataset congruence metrics for parsimony trees (equal weights) and Bayesian trees for each metric. Linear regressions (red lines) are tested for their divergence from 1:1 relationship (blackline); SCI, GER, and MSM* all show a tendency for relatively congruent datasets to exhibit elevate congruence for parsimony trees (*p*=2.3x10^-6^, 4.2x10^-8^ and 2x10^-16^ for regression slope different from 1 respectively).
